# A spike in circulating cytokines TNF-α and TGF-β alters barrier function between vascular and musculoskeletal tissues

**DOI:** 10.1038/s41598-023-30322-7

**Published:** 2023-06-05

**Authors:** Lucy Ngo, Melissa L. Knothe Tate

**Affiliations:** 1 MechBio Team, Graduate School of Biomedical Engineering, University of New South Wales, Sydney, Australia; 2 Blue Mountains World Interdisciplinary Innovation Institute, New South Wales, Australia

**Keywords:** Biological techniques, Biotechnology, Immunology, Physiology, Systems biology, Diseases, Rheumatology, Engineering, Nanoscience and technology, Optics and photonics

## Abstract

Molecular transport between the circulatory and musculoskeletal systems regulates articular joint physiology in health and disease. Osteoarthritis (OA) is a degenerative joint disease linked to systemic and local inflammation. Inflammatory events involve cytokines, which are secreted by cells of the immune system and modulate molecular transport across tissue interfaces (referred to as *tight junction [TJ] barrier function*). In a previous study from our group, OA knee joint tissues were shown to exhibit size separation of different sized molecules delivered as a single bolus to the heart (Ngo et al. in Sci. Rep. 8:10254, 2018). Here, in a follow up study of parallel design, we test the hypothesis that two common cytokines, with multifaceted roles in the etiology of osteoarthritis as well as immune state in general, modulate the barrier function properties of joint tissue interfaces. Specifically, we probe the effect of an acute cytokine increase (spike) on molecular transport within tissues and across tissue interfaces of the circulatory and musculoskeletal systems. A single bolus of fluorescent-tagged 70 kDa dextran, was delivered intracardially, either alone, or with either the pro-inflammatory cytokine TNF-α or the anti-inflammatory cytokine TGF-β, to skeletally mature (11 to 13-month-old) guinea pigs (Dunkin-Hartley, a spontaneous OA animal model). After five minutes' circulation, whole knee joints were serial sectioned and fluorescent block face cryo-imaged at near-single-cell resolution. The 70 kDa fluorescent-tagged tracer is analogous in size to albumin, the most prevalent blood transporter protein, and quantification of tracer fluorescence intensity gave a measure of tracer concentration. Within five minutes, a spike (acute doubling) in circulating cytokines TNF-α or TGF-β significantly disrupted barrier function between the circulatory and musculoskeletal systems, with barrier function essentially abrogated in the TNF-α group. In the entire volume of the joint (including all tissue compartments and the bounding musculature), tracer concentration was significantly decreased in the TGF-β- and TNF-α- compared to the control-group. These studies implicate inflammatory cytokines as gatekeepers for molecular passage within and between tissue compartments of our joints and may open new means to delay the onset and mitigate the progression of degenerative joint diseases such as OA, using pharmaceutical and/or physical measures.

## Introduction

Joint tissues, including cartilage, synovium, bone, subchondral bone, periarticular musculature, meniscus, ligament, and other connective tissues, form an integrated biosystem. Musculoskeletal cells rely for their survival on the movement of molecules, from the cardiovascular system to and between vascularized and avascular tissue compartments within the joint^1–3^. A recent study from our group showed musculoskeletal tissue compartments exhibit molecular size selectivity, suggesting the presence of functional barrier properties at tissue interfaces such as the periosteum and synovial membrane, and implicating age-related changes to molecular transport in OA pathogenesis^4^. Albumin (67 kDa), the main carrier protein in blood plasma, presents an upper bound for molecular size permeability in bone tissue in absence of mechanical loading^5^. It is currently unknown to what degree albumin and larger proteins permeate tissue compartments of the synovial joint complex.

While the underlying mechanisms of OA are poorly understood, there is growing evidence that changes in molecular transport within and between tissue compartments, brought on by systemic inflammation, may underpin or at least predispose patients to OA^6–8^. OA has long been attributed as a disease of cartilage alone. Current paradigms now consider OA as a condition of the entire joint, with the breakdown of cellular communication between tissues of the joint central to OA’s pathogenesis and progression^9^. To date, few studies have addressed the tissue interface's role as a gatekeeper for molecular transport between the cardiovascular and musculoskeletal systems. A multiscale approach, *i.e.* sub cell to tissue to organ, may be particularly useful to understand the interplay of chronic systemic inflammation typical for OA, acute inflammatory cytokine spikes associated with trauma or bacteria/virus exposure, and molecular transport within and between tissue compartments of the joint. This represents an important but neglected gap in knowledge in the field and may play an important role in understanding the pathogeneses of OA as well as other degenerative joint diseases.

Chronic low-grade systemic inflammation is characterized by sustained, heightened levels of inflammatory cytokines. Increases in cytokines, including tumor necrosis factor alpha (TNF-α) and transforming growth factor beta (TGF-β), relate to both systemic inflammation and age-related degenerative diseases, including osteoarthritis^10–12^. The pro-inflammatory cytokine TNF-α and the anti-inflammatory cytokine TGF-β have been shown to modulate molecular transport dynamics at interfaces between the circulatory system and diverse tissues by controlling paracellular permeability through regulation of tight junction (TJ) protein complexes^13^. Acute inflammation due to trauma and bacteria/virus exposure results in cytokine spikes.

Observation of relative changes in molecular transport, to and between the circulatory system and joint tissues, in response to dynamic cytokine concentrations (systemic as well as local), and facilitated by blood and interstitial fluid flow, requires multiscale imaging from the sub-micro to mesoscale, across cells, tissues, and the joint organ. Here we hypothesized that acute changes to immune modulatory cytokines alter functional barrier properties at tissue interfaces between the tissues of the musculoskeletal and the circulatory systems. To test this hypothesis, we quantified changes to molecular transport within and between the tissue compartments of the osteoarthritic joint, using a parallel approach to our previous study, *i.e.* in anaesthetized Guinea pigs injected via the heart with a tagged 70 kDa molecular tracer and the cytokines TNF-α or TGF-β^3,14–16^.

## Results and discussion

Our overarching aim was to to understand global and local differences in perfusion to the tissues of the knee after an acute spike in either the respective pro-inflammatory or anti-inflammatory cytokines, TNF-α or TGF-β. First, the mean tracer intensity was measured within the entire tissue block of each animal within each group; this bulk measure of knee joint perfusion included the joint and surrounding musculature. Thereafter, mean tracer intensity was compared between tissue compartments of the joint, within and between groups (cytokine treated and untreated control). Finally, barrier function at the vascular interface within tissues of the joint was measured as the difference between the vascular and tissue compartments of bone and muscle tissue.

The permeability of large molecules such as albumin was measured using a 70 kDa fluorescent molecular tracer, where the tracer intensity is directly proportional to concentration^17^. A decrease in tracer intensity indicates a decreased tracer concentration and thus an decrease in tracer permeability; conversely, an increase in tracer intensity indicates an increased tracer concentration and thus an increase in tracer permeability. Within each animal group (two cytokine treatment groups—TNF-α or TGF-β—and untreated control group), relative differences in tracer concentration between tissue compartments give a measure of barrier function between respective tissues.

### Cytokines modulate permeability of large molecules in the entire joint

**Figure 1:**
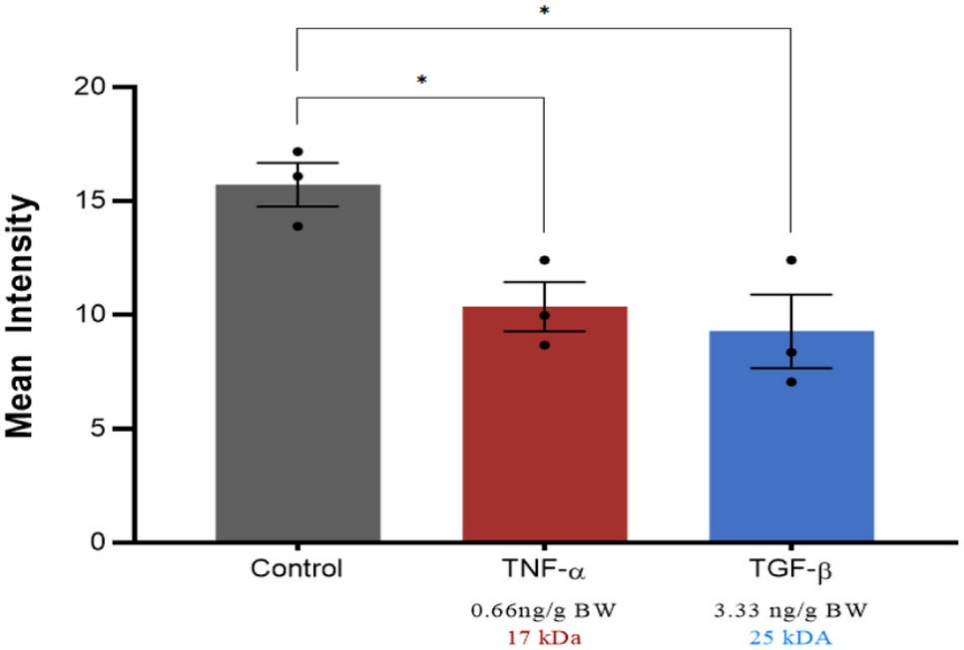
Mean 70 kDa tracer intensity in the entire tissue block containing the joint and surrounding musculature. Error bars indicate standard error of the mean (n = 3). *Significant differences were determined by one-way ANOVA with Tukey’s post hoc analysis, where statistical significance is defined by *p* < 0.05.

To test the hypothesis that co-delivery of the 70 kDa tracer with cytokine (TNF-α or TGF-β) modulates molecular transport (permeability or tissue penetration), mean tracer intensity was first compared between the control and each of the experimental cytokine groups using a one-way ANOVA with a Tukey's post-hoc tests (Fig. 1). The TGF-β and the TNF-α groups exhibited a significantly lower mean intensity than the control group (*p* < 0.05). No significant differences were observed between the cytokine treatment groups.

### A spike in circulating cytokines disrupts tissue barrier function between muscle and the joint

**Figure 2:**
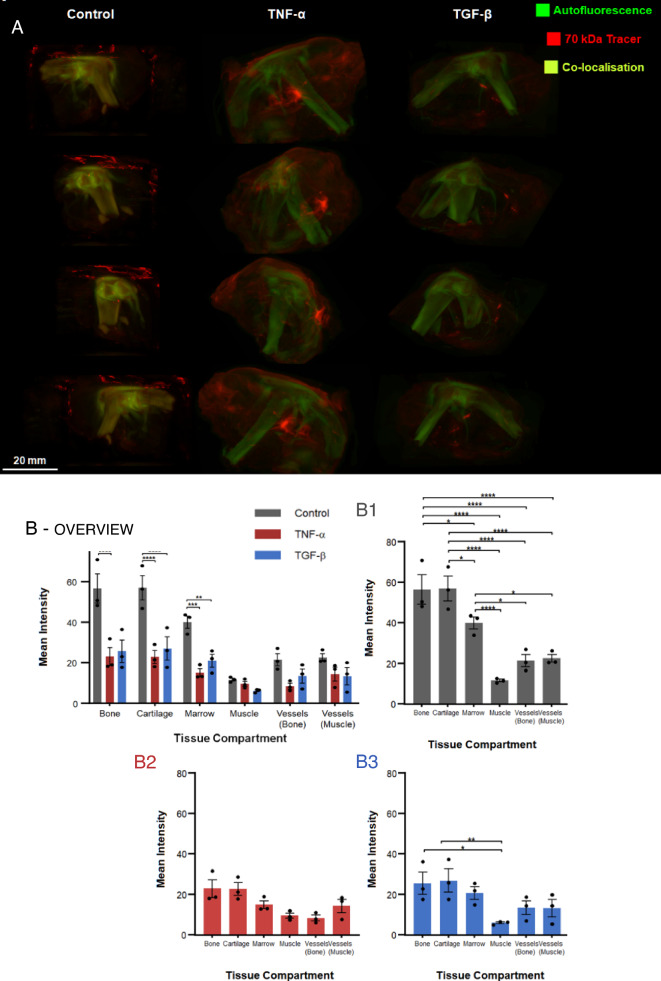
An acute increase in circulating cytokines disrupts barrier function between tissue compartments compared to controls. Barrier function is highest between tissue compartments with highest (significant) differences in tracer concentration. (**A**) 3D rendering showing gross tracer distribution within the joint. In the control group (left panel), tracer intensity was markedly higher within the bone, cartilage and marrow space compared to the surrounding musculature, with tracer also appearing in ligaments. The cartilage, bone and ligaments appeared yellow-green due to co-localization of the tracer (exogenous fluorescence, red) and autofluorescence of the tissue (endogenous fluorescence of collagen, green). The surrounding musculature exhibited little to no fluorescence, with the bounding muscle fasciae exhibiting somewhat elevated levels of tracer. In contrast to both the control group and TGF-β group, the TNF-α group (middle panel) showed highest grossly apparent levels of tracer, both in the bounding muscle fasciae which starkly delineate the muscle compartment, as well as in the muscle vasculature. Also, in contrast to the control group, the TNF-α and TGF-β groups exhibited less tracer in cartilage, ligament, and bone, which appeared almost exclusively green (i.e. exhibiting autofluorescence but little to no evidence of red tracer). Similarly, low levels of tracer were observed within the marrow space. The TGF-β group exhibited similar patterns of tracer albeit much less red fluorescence intensity in muscle (right panel). Tissue autofluorescence (green) and fluorescent tagged 70 kDa tracer (red) give a yellow signal when colocalized. (**B**-overview) Comparison of mean tracer concentration across tissue compartments in all groups. A spike in TNF-α results in a significant decrease in tracer concentration in bone, cartilage and marrow. A spike in TGF-β results in a significant decrease in tracer concentration in cartilage and marrow. Taking each specific group into consideration, including the control (**B1**), TNF-α (**B2**) and TGF-β (**B3**) groups, more significant differences in tracer concentration are observed between tissue compartments of the control group (**B1**) than the experimental groups (**B2**, **B3**). Compared to the intact functional barriers at interfaces between tissues of the control group, barrier function appears reduced in the TGF-β group and completely abrogated in the TNF-α group. Error bars indicate standard error (n = 3). Shapiro–Wilk tests showed that the quantitative data was normally distributed. Significant differences were determined by two-way ANOVA and Sidak’s multiple comparisons post hoc analysis. Statistical significance indicated respectively by *, **, ***, **** (*p* < 0.05, *p* < 0.01, *p* < 0.001 and *p* < 0.001).

Three-dimensional (3D) renderings of the entire joint volume (Fig. 2A) showed gross relative differences in distribution of the 70 kDa tracer. These were confirmed through quantitative measure of the mean tracer intensity in different tissue compartments of the joint, and between extra- and intravascular compartments of respective tissues, which give quantitative measures of tissue barrier function.

In the control group, tracer concentrations were highest (not significantly different) in the bone and cartilage compartments. The bone and cartilage compartments of the control knee joints both exhibited significantly higher tracer concentration than bone marrow and the bone marrow compartment showed significantly higher tracer concentration than the muscle and the respective vascular compartments of the bone and muscle. Taken together, these significant differences in respective tracer concentrations between tissue compartments of the control knee indicate an intact, baseline barrier function between joint tissues of the untreated control group (Fig. 2B1).

Within groups, differences in tracer concentration between tissue compartments give a measure of barrier function between respective tissue compartments. To test the hypothesis that co-delivery of the tracer with the cytokine, TNF-α or TGF-β, alters the amount and the spatial distribution of tracer within the different tissue compartments of the joint, surrounding musculature, and vascular system, tracer concentrations were compared between segmented tissue compartments of the control, TNF-α and TGF-β groups. In the control group, the bone, cartilage, and bone marrow tissue compartments showed significantly higher tracer concentration than the respective tissue compartments of both the TNF-α and TGF-β groups, confirming qualitative, macroscopic observations (Fig. 2B1). Furthermore, respective tissue compartments of the control group showed more statistically significant differences in tracer concentration than those of the TGF-β group (Fig. 2B3), and the TGF-β group showed more statistically significant concentration differences than in the respective compartments of the TNF-α group (Fig. 2B2).

Hence, these data implicate co-delivery of the cytokines, TNF-α and TGF-β, in changing the concentration and distribution of tracer in tissues of the joint such that differences between tissues are reduced, implicating loss in barrier function between tissue compartments. In the TGF-β group, barrier function was reduced compared to the control group. In the TNF-α group, barrier function appeared to be completely abrogated, as there were no significant differences in tracer concentration between tissue compartments of that group.

### Modulation of large molecule permeability at vasculature-tissue interfaces

**Figure 3:**
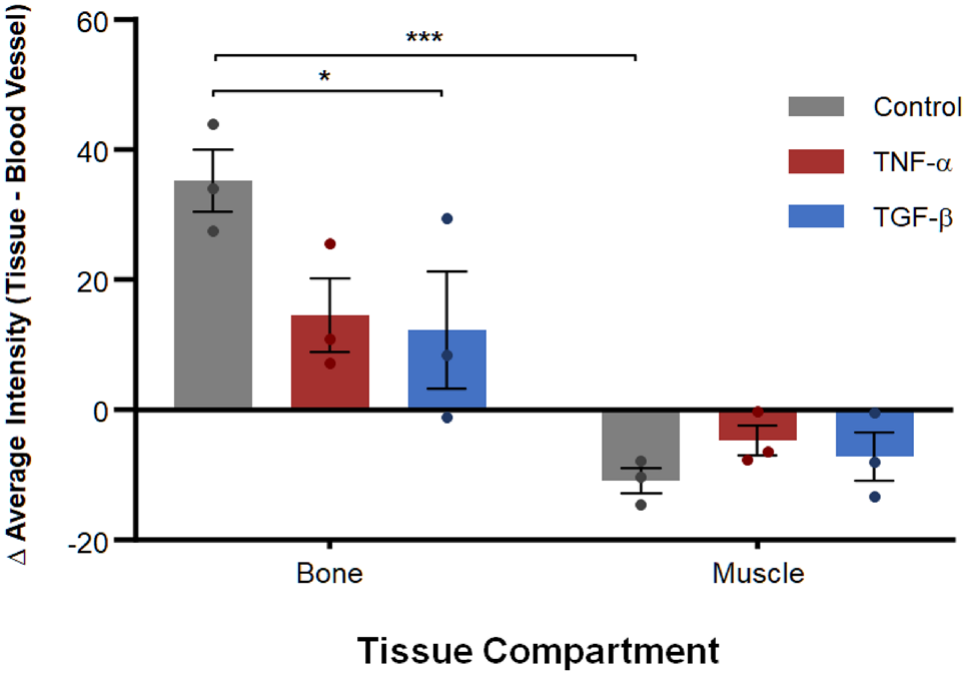
Barrier function at the vascular interface with tissue compartments including bone and muscle, in control as well as TNF-α or TGF-β groups. Bone and muscle exhibit significant magnitude and directional differences in barrier function for the control group but not for the TNF-α or TGF-β groups, indicating a reduction in barrier function for both cytokine groups. Further, significant differences in barrier function between bone’s vascular and tissue compartments were observed between the control and the TGF-β group, indicating a significant decrease in barrier function between blood vessels and bone with delivery of TGF-β. Error bars indicate standard error (n = 3). Significant differences were determined by two-way ANOVA and Sidak’s multiple comparisons post hoc analysis. Statistical significance indicated by * (*p* < 0.05) and *** (*p* < 0.001).

To test the hypothesis that co-delivery of tracer with cytokines, TNF-α or TGF-β, changes barrier function of interface tissues including periosteum (between bone and muscle) and vascular endothelium (intra- *versus* extravascular compartments), the difference in mean tracer concentration was compared between the extravascular tissue compartments of bone and muscle and their respective intravascular compartments (blood vessels). Acute delivery of TNF-α or TGF-β was shown to reduce barrier function at tissue-vessel interfaces. Specifically, differences in intra- to extravascular 70 kDa tracer transport in bone were significantly greater in the control group than in the TGF-β group, indicative of intact barrier function. Additionally, in the control group, barrier function in bone was significantly greater and in an opposite direction in bone than in muscle. In contrast, the groups injected with TNF-α or TGF-β did not show a significant difference in barrier function as measured between intra- and extravascular transport in tissue compartments (Fig. 3).

This study investigated whether spikes in immune modulating cytokines, TNF-α or TGF-β, induce changes to tissue barrier function at tissue interfaces within the knee joint of guinea pigs with spontaneous, age-related OA, using near single cell resolution 3D cryo-imaging of entire joints 5 min after cytokine and fluorescent-tagged tracer delivery to the heart. Stark, grossly observable changes in the fluorescence-tagged, 70 kDa tracer distribution were observable in macroscopic renderings of the periarticular muscle compartments, as well as the cartilage, bone, and marrow space of the joint complex. In the control group, quantification of tracer concentration within tissue compartments of the bone, cartilage, marrow, and muscle compartments, as well as their respective vascular compartments, demonstrated significant differences between multiple compartments, indicative of intact barrier function at inter-compartmental interfaces. In the TGF-β group, respective barrier function appeared reduced. Barrier function appeared to be completely abrogated in the TNF-α group, as no significant differences in tracer concentrations were observed between any tissue compartments measured. Furthermore, significant differences in magnitude and direction of tracer transport were observed between the vascular and the extravascular compartments of bone and muscle of the control group, indicative of intact barrier function and corroborating previous studies of periosteum' role as a molecular gatekeeper between bone and muscle^18^.

TNF-α and TGF-β dosages administered in the current experiment represent an acute increase (spike) in cytokine levels **above** the intrinsic, chronic (patho-)physiological levels attributable to the osteoarthritic disease state of a given animal. The equivalent spike in serum levels of TNF-α and TGF-β (5 min after administration to the heart) were calculated to be 16.5 ng/ml and 83.25 ng/ml, respectively. Baseline TNF-α and TGF-β serum levels in healthy and OA guinea pigs have not yet been reported. Cytokine serum levels have been characterized in induced rodent models of OA; the serum levels of TNF-α administered in the current experiment represent double the baseline levels of the murine OA model, and the serum levels of TGF-β comprise 62% of reported values^19,20^. Hence, the effect of cytokine administration in the current study represents a near immediate doubling of TNF-α and TGF-β over baseline levels. Taking this into account for the guinea pig model, a near immediate doubling of TNF-α over baseline levels appeared to completely abrogate barrier function between tissues, whereas as an acute circa 62% increase in TGF-β mitigated barrier function between tissues to a lesser degree over baseline barrier function (control group). In other words, a higher spike in concentration of cytokine over baseline levels was associated with a bigger impact on, i.e. decrease, in barrier function between tissues.

Published literature reports an increase in vascular albumin permeability with exogenously administered TNF-α in studies carried out in an in vivo mouse model investigating multiple tissues including lung, liver, kidney, brain, sciatic nerve, retina, duodenum, jejunum, cecum, thoracic aorta, skin and diaphragm^21^. In contrast to the current acute study observing changes 5 min after TNF-α delivery, significant increases were not observed in the mouse study until 12–18 h after delivery of TNF-α. Furthermore, the published mouse study administered TNF-α at 20 ng/g, a greater than 30-fold increase in dose compared to the current study. These differences may also reflect intrinsic differences between the healthy murine model and the natural model of OA in the guinea pig.

In the current study, the TNF-α and TGF-β group exhibited significantly lower concentrations of tracer in the muscle and joint tissues, indicative of decreased permeability; this contrasts with published in vivo studies where reduction in TGF-β activity decreased permeability of the blood brain barrier^22^. However, TGF-β has been shown to both enhance and disrupt in vitro barrier function in intestinal epithelial cells, brain microvascular cells, colonic epithelial cells, endothelial and corneal endothelial cells, and alveolar epithelial cells and Sertoli cells, respectively^23–28^. In oral epithelial cells, TGF-β has been shown to decrease and then to increase functional barrier permeability^29^. In vivo experiments have demonstrated increased systemic tissue permeability to small molecules, 1.8 kDa, after fracture^30^. Elevated levels of serum TGF-β have also been detected in response to fracture^31^. Taken as a whole, the effect of TGF-β functional barrier modulation may be tissue, time, and molecular size dependent.

Bone-muscle crosstalk, mediated by the periosteum, has been associated with other age-related degenerative tissue conditions, namely sarcopenia and osteoporosis^32,33^. However, its role in osteoarthritis has not been as widely recognized. Periosteum serves as a functional barrier membrane enveloping bone and as a stimuli-responsive interface between bone and muscle^18,34–36^. Of particular note, the barrier function of periosteum has been shown previously to be direction dependent, with permeability in the bone to muscle direction significantly less than in the muscle to bone direction; these effects are stimuli-responsive to change in flow rate as well as prestress, both of which are immediate indicators of trauma^18,35,37^. In context of the current study, a loss in barrier function attributable to a rapid spike in cytokines TNF-α and TGF-β would amplify signaling via molecular transport from muscle to bone and vice versa*.* Hence periosteum provides a key interface between the circulatory system and the joint. A comparison of tissue compartments reveals that transport of the 70 kDa molecule to bone, cartilage, and marrow space is significantly disrupted by TNF-α and TGF-β; in contrast the muscle and vasculature compartments remain largely unaffected. This suggests the periosteum actively modulates the movement of macromolecules between muscle and bone in response to inflammatory cytokines, implicating it as a functional tissue barrier, further reinforced by published observations of tight junction associated protein expression by periosteum-derived cells^34^.

It has been hypothesized that perturbations to vascular function relate to the onset or progression of OA, with insufficient blood flow causing cell death throughout the tissues of the joint^38^. In the control animals, a comparison of bone and muscle, and their respective vasculature, reveal significantly greater levels of mean tracer intensity in bone than in muscle. This suggests the endothelial barrier within bone has greater active barrier properties than in muscle and/or that relative lymphatic joint-drainage effects are potentially greater in muscle than in bone tissue (see discussion below). The permeability of this endothelial barrier within bone vasculature is also significantly affected by both TNF-α and TGF-β, implicating increases in inflammatory cytokines in vascular barrier dysfunction. The associated disruption in transport of nutrients, cytokines and proteolytic enzymes to cells and the extracellular matrix of bone and cartilage, and the associated disruptions to tissue homeostasis, may represent a contributing mechanism by which low grade systemic inflammation leads to tissue degeneration and OA pathogenesis.

It was not possible in the current study to examine the concentration of tracer in lymphatic vessels, which may change their joint-drainage capacity in response to circulating cytokines^39–41^. Lymphatic drainage has been shown to increase and decrease, respectively at the initial onset and with the chronic progression of arthritis, where persistent inflammation is associated in destruction of lymph vessel structure and function^40^. In the current study, the guinea pigs are a spontaneous model of OA, so their tissues are likely to exhibit both features of chronic disease (with associated decreases in lymphatic drainage) as well as exposure to an acute spike in cytokines TGF-β or TNF-α (with associated increase in lymphatic drainage); furthermore, the specificity of human TNF-α and TGF-β towards guinea pig equivalents is unknown. One must also consider that lymphatic vessels are abundant in muscle while bone relies on lymphatic drainage through periosteal tissue^18,36^. With the development of new methodologies, it may be possible in the future to decipher interrelated effects of barrier function between the vascular and musculoskeletal system concomitant to lymphatic drainage function.

A further limitation to the current study is that, although the selected dextran is of similar molecular weight to albumin (70 kDa compared to 67 kDa albumin), the dextran is linear unlike the globular structure of the carrier protein albumin. Molecular transport is affected by shape, with linear molecules demonstrating higher hydrodynamic radii and resulting in slower diffusion than globular molecules. Hence, transport of the 70 kDa tagged dextran “biological analog” may represent a higher estimate of equivalent transport of the 67 kDa globular albumin molecules, albeit somewhat counterbalanced by the slightly higher molecular mass and the capacity to “worm through” smaller openings. Furthermore, although fluorescent tagged dextran used in the current study is of neutral charge, the molecule is polar as it is labelled “zwitterionic”; in contrast, albumin is negatively charged. Molecular charge has also been shown to impact molecular transport; however, it has been shown that charge does not affect diffusion of large molecules (66 kDa) in tissues^42^. In addition, block face cryo-imaging enables unprecedented 3D seamless imaging of transport pathways within and between tissue compartments and systems of the organism across multiple length scales, albeit at a single time point in a highly dynamic process. This is an advantage for the current study, assessing effects of a near-immediate increase in circulating cytokines. Follow on longitudinal and in vivo (*e.g.* with near real time imaging modalities^3,32,43^), experiments would increase understanding of transport dynamics as well as the effects of acute to chronic cytokine increases, mimicking cytokine storms to systemic inflammation, on the temporal unfolding of molecular traffic patterns between the circulatory and musculoskeletal systems.

The present study further implicates acute changes in cytokines for near immediate changes in barrier function at interfaces between tissues of the circulatory and musculoskeletal systems. It may be possible in the future to harness this reduction in barrier function to target delivery of pharmaceuticals to the inhabitant cells, within cartilage (chondrocytes) and bone (osteoblasts, osteocytes), to repair and rebuild tissues ravaged by degenerative diseases such as OA^44^. Although temporal effects were not specifically addressed by the current study, against a backdrop of low-grade systemic inflammation in OA, the acute increase in cytokines TNF-α and TGF-β appears to immediately disrupt barrier function at tissue and vascular interfaces. Further, the study demonstrates the immune-modulation capacity of barrier properties that exist between tissue compartments, extending the role of crosstalk in OA from cartilage and subchondral to all the tissues within the joint. It is currently unknown how mechanical loading and active transport alter molecular transport within joint tissues in response to inflammatory cytokines. A recent study suggests improved efficacy with "direct delivery" of pharmaceuticals to the local joint lymphatics^41^. Taken as a whole, these studies may provide new opportunities to understand joint physiology and tissue degeneration, and to develop pharmaceutical and physical measures to prevent disease onset and progression.

## Methods

Here we studied the effect of acute increases in inflammatory cytokines, on transport of a 70 kDa tagged dextran from the cardiovascular system to and between the tissue compartments of the joint. a full protocol of methodologies described in brief below can be found at^45^.

### Choice of molecular tracer and animal model

The 70 kDa tagged dextran was selected based on previous experiments showing molecular sieving in bone, ligament and the entire joint; additionally it serves as an analog for large molecules such as albumin (67 kDa), the most common carrier protein in the blood and fundamental to facilitating this transport^1,3–5,46–48^. The Dunkin-Hartley guinea pig is a well-established model for spontaneous age-related OA of the knee and other synovial joints, with pathophysiological processes mirroring the primary OA in humans^3,4,14–16^. Experiments were performed on skeletally mature (11–13 months) male Dunkin-Hartley guinea pigs (Charles River), shown in a previous study from our group to exhibit OA phenotype including multiple osteophytes as well as large areas of cartilage loss with marked bone-on-bone cartilage erosion^4^.

### Bolus preparation and administration

Preparation, administration, and five minutes' circulation time of the fluorescent tracer was carried out per previously described methods^4,5,48^. Recombinant human TNF-α (R and D Systems, Minneapolis, MN) was dissolved in 0.9% sterile saline solution and added to the mixed bolus at a dose of 0.66 ng/g body weight prior to administration to the heart of anesthetized Guinea pigs. Recombinant human TGF-β1 (R & D Systems, Minneapolis, MN; referred to as TGF-β in the text for simplicity) was similarly prepared at a dose of 3.33 ng/g body weight.

### Animal preparation

This study was conducted in accordance with ARRIVE guidelines and regulations. The methods were conducted in accordance with the relevant guidelines and regulations. The study was approved by the Institutional Animal Care and Use Committee (IACUC) at Case Western University (Protocol #2012-0100). Three groups of animals (n = 3) were anesthetized with isoflurane; after appropriate sedation, a 70 kDa Texas-red tagged dextran of neutral charge (Life Technologies, Carlsbad) alone (control) or with TNF-α or TGF-β, was injected via the heart as a single mixed bolus. After allowing five minutes’ circulation, the animals were euthanized while still under anesthesia. Post euthanasia, the knee joints were immediately resected, then proximally and distally osteotomized, with care taken to not damage the surrounding musculature. The joint was then flash frozen by liquid nitrogen immersion and embedded optical cutting temperature compound for high resolution cryo-imaging.

### Cryo-fluorescence Imaging

The entire OCT-embedded tissue blocks were sectioned and imaged every 40 μm with the CryoViz™ automated block face cryo-imaging system (BioInvision Inc., Cleveland, OH), enabling three-dimensional near-single cell resolution of fluorophores in mesoscopic specimens^49^. Brightfield and fluorescence (502–543 nm and 603–664 nm) images were acquired at 10.2 × 10.2 μm in plane resolution. Tissue compartments were manually segmented and the mean fluorescence intensity of the region of interest was used as a measure for tracer permeation.

### Statistical analysis

GraphPad Prism 8 (GraphPad Software, San Diego California USA) was used for statistical analysis, with reporting of means and standard errors. We used a one-way ANOVA statistical analysis and Tukey's post hoc analysis for comparisons between multiple tissue compartments. *P* < 0.05 was considered significant.

## Data Availability

The datasets generated and/or analysed during the current study are available in the MechBio repository, https://mechbio.org.

## References

[1] Knothe Tate, M.L. & Niederer, P. A theoretical FE-based model developed to predict the relative contribution of convective and diffusive transport mechanisms for the maintenance of local equilibria within cortical bone. in *Advances in Heat and Mass Transfer in Biotechnology*, Scott Clegg, Ed. HTD – Vol. 362/BED – Vol. 40, *Advances in Heat and Mass Transfer in Biotechnology*, (Ed. S. Clegg) The American Society of Mechanical Engineers, 133–142. (1998).

[2] Knothe Tate ML, Niederer P, Knothe UR (1998). In vivo tracer transport through the lacunocanalicular system of rat bone in an environment devoid of mechanical loading. Bone.

[3] Knothe Tate, M.L., Aaron, R.K., Ignatius, A., Dürselen, L., Rockson, S. Molecular tranport in musculoskeletal health and disease, In: Aaron R.K. (eds) *Skeletal Circulation in Clinical Practice.* World Scientific, Singapore (2016).doi:10.1142/9746.

[4] Ngo L, Knothe LE, Knothe Tate ML (2018). Knee joint tissues effectively separate mixed sized molecules delivered in a single bolus to the heart. Sci. Rep..

[5] Tami AE, Schaffler MB, Knothe Tate ML (2003). Probing the tissue to subcellular level structure underlying bone’s molecular sieving function. Biorheology.

[6] Sokolove J, Lepus CM (2013). Role of inflammation in the pathogenesis of osteoarthritis: Latest findings and interpretations. Ther. Adv. Musculoskelet. Dis..

[7] Scanzello CR, Loeser RF (2015). Editorial: Inflammatory activity in symptomatic knee osteoarthritis: Not all inflammation is local. Arthr. Rheumatol..

[8] Mathiessen A, Conaghan PG (2017). Synovitis in osteoarthritis: Current understanding with therapeutic implications. Arthr. Res. Ther..

[9] Brandt KD, Radin EL, Dieppe PA, van de Putte L (2006). Yet more evidence that osteoarthritis is not a cartilage disease. Ann. Rheum. Dis..

[10] Müller B (2002). Cytokine imbalance in non-immunological chronic disease. Cytokine.

[11] Deligne C, Casulli S, Pigenet A, Bougault C, Campillo-Gimenez L, Nourissat G, Berenbaum F, Elbim C, Houard X (2015). Differential expression of interleukin-17 and interleukin-22 in inflamed and non-inflamed synovium from osteoarthritis patients. Osteoarthr. Cartil..

[12] Rea, I.M., Gibson, D.S., McGilligan, V., McNerlan, S.E., Alexander, H.D., & Ross, O.A. Age and age-related diseases: Role of inflammation triggers and cytokines. *Front. Immunol.* **9**, 586 (2018). 10.3389/fimmu.2018.00586 PMC5900450 29686666

[13] Al-Sadi R, Boivin M, Ma T (2009). Mechanism of cytokine modulation of epithelial tight junction barrier. Front. Biosci. (Landmark Ed.).

[14] Jimenez PA, Glasson SS, Trubetskoy OV, Haimes HB (1997). Spontaneous osteoarthritis in Dunkin Hartley guinea pigs: Histologic, radiologic, and biochemical changes. Lab. Anim. Sci..

[15] Huebner JL, Hanes MA, Beekman B, TeKoppele JM, Kraus VB (2002). A comparative analysis of bone and cartilage metabolism in two strains of guinea-pig with varying degrees of naturally occurring osteoarthritis. Osteoarthr. Cartil..

[16] Aaron RK, Dyke JP, Ciomber DM, Ballon D, Lee J, Jung E, Tung GA, Ciombor DM, Ballon D, Lee J, Jung E, Tung GA, Ciomber DM, Ballon D, Lee J, Jung E, Tung GA (2007). Perfusion abnormalities in subchondral bone associated with marrow edema, osteoarthritis, and avascular necrosis. Ann. N. Y. Acad. Sci..

[17] Lee K, Shirshin E, Rovnyagina N, Yaya F, Boujja Z, Priezzhev A, Wagner C (2018). Dextran adsorption onto red blood cells revisited: Single cell quantification by laser tweezers combined with microfluidics. Biomed. Opt. Express..

[18] Evans SF, Parent JB, Lasko CE, Zhen X, Knothe UR, Lemaire T, Knothe Tate ML (2013). Periosteum, bone’s ‘smart’ bounding membrane, exhibits direction-dependent permeability. J. Bone Miner. Res..

[19] Yu F, Xie C, Jiang C, Sun J, Huang X (2018). TNF-α increases inflammatory factor expression in synovial fibroblasts through the toll-like receptor-3-mediated ERK/AKT signaling pathway in a mouse model of rheumatoid arthritis. Mol. Med. Rep..

[20] Waly NE, Refaiy A, Aborehab NM (2017). IL-10 and TGF-β: Roles in chondroprotective effects of Glucosamine in experimental Osteoarthritis?. Pathophysiology.

[21] Worrall NK, Chang K, Lejeune WS, Misko TP, Sullivan PM, Ferguson TB, Williamson JR (1997). TNF-α causes reversible in vivo systemic vascular barrier dysfunction via NO-dependent and -independent mechanisms. Am. J. Physiol. Circ. Physiol..

[22] McMillin MA, Frampton GA, Seiwell AP, Patel NS, Jacobs AN, DeMorrow S (2015). TGFβ1 exacerbates blood-brain barrier permeability in a mouse model of hepatic encephalopathy via upregulation of MMP9 and downregulation of claudin-5. Lab. Invest..

[23] Planchon SM, Martins CA, Guerrant RL, Roche JK (1994). Regulation of intestinal epithelial barrier function by TGF-beta 1. Evidence for its role in abrogating the effect of a T cell cytokine. J. Immunol..

[24] Dohgu S, Yamauchi A, Takata F, Naito M, Tsuruo T, Higuchi S, Sawada Y, Kataoka Y (2004). Transforming growth factor-beta1 upregulates the tight junction and P-glycoprotein of brain microvascular endothelial cells. Cell. Mol. Neurobiol..

[25] Howe KL, Reardon C, Wang A, Nazli A, McKay DM (2005). Transforming growth factor-beta regulation of epithelial tight junction proteins enhances barrier function and blocks enterohemorrhagic Escherichia coli O157:H7-induced increased permeability. Am. J. Pathol..

[26] Beaulieu Leclerc V, Roy O, Santerre K, Proulx S (2018). TGF-β1 promotes cell barrier function upon maturation of corneal endothelial cells. Sci. Rep..

[27] Lui W-Y, Lee WM, Cheng CY (2001). Transforming growth factor-β3 perturbs the inter-sertoli tight junction permeability barrier in vitro possibly mediated via its effects on Occludin, Zonula Occludens-1, and Claudin-11 1. Endocrinology.

[28] Pittet J-F, Griffiths MJD, Geiser T, Kaminski N, Dalton SL, Huang X, Brown LAS, Gotwals PJ, Koteliansky VE, Matthay MA, Sheppard D (2001). TGF-β is a critical mediator of acute lung injury. J. Clin. Invest..

[29] Ye P (2012). Modulation of epithelial tight junctions by TGF-beta 3 in cultured oral epithelial cells. Aust. Dent. J..

[30] Tami, A., Schaffler, M.B., & Knothe Tate, M.L. Cellular and Tissue-Level Permeability Are Increased in Fractured and in Contralateral Control Bones. in *Proceedings of the ASME 2001 Summer Bioengineering Conference* 189–190 (American Society of Mechanical Engineers, 2001).

[31] Sarahrudi K, Thomas A, Mousavi M, Kaiser G, Köttstorfer J, Kecht M, Hajdu S, Aharinejad S (2011). Elevated transforming growth factor-beta 1 (TGF-β1) levels in human fracture healing. Injury.

[32] Elphingstone, J., Hamrick, M.W. Muscle and Bone Biology – Similarities and Differences. In: Duque G. (eds) *Osteosarcopenia: Bone, Muscle and Fat Interactions*. Springer, Cham (2019). 10.1007/978-3-030-25890-0_1

[33] Maurel D, Jähn K, Lara-Castillo N (2017). Muscle-bone crosstalk: Emerging opportunities for novel therapeutic approaches to treat musculoskeletal pathologies. Biomedicines.

[34] Evans SF, Docheva D, Bernecker A, Colnot C, Richter RP, Knothe Tate ML (2013). Solid-supported lipid bilayers to drive stem cell fate and tissue architecture using periosteum derived progenitor cells. Biomaterials.

[35] McBride SH, Dolejs S, Brianza S, Knothe U, Knothe Tate ML (2011). Net change in periosteal strain during stance shift loading after surgery correlates to Rapid De Novo Bone generation in critically sized defects. Ann. Biomed. Eng..

[36] Evans SF, Chang H, Knothe Tate ML (2013). Elucidating multiscale periosteal mechanobiology: A key to unlocking the smart properties and regenerative capacity of the Periosteum?. Tissue Eng. Part B Rev..

[37] Yu NYC, Orien CA, Slapetova I, Whan RM, Knothe Tate ML (2017). Live tissue imaging to elucidate mechanical modulation of stem cell niche quiescence. Stem Cells Transl. Med..

[38] Findlay DM (2007). Vascular pathology and osteoarthritis. Rheumatology.

[39] Scallan JP, Zawieja SD, Castorena-Gonzalez JA, Davis MJ (2016). Lymphatic pumping: Mechanics, mechanisms and malfunction. J. Physiol..

[40] Bouta EM, Bell RD, Rahimi H, Xing L, Wood RW, Bingham CO, Ritchlin CT, Schwarz E (2018). Targeting lymphatic function as a novel therapeutic intervention for rheumatoid arthritis. Nat. Rev. Rheumatol..

[41] Lam AD, Cao E, Leong N, Gracia G, Porter CJH, Feeney OM, Trevaskis NL (2022). Intra-articular injection of biologic anti-rheumatic drugs enhances local exposure to joint-draining lymphatics. Eur. J. Pharm. Biopharm..

[42] Bajpayee AG, Wong CR, Bawendi MG, Frank EH, Grodzinsky AJ (2014). Avidin as a model for charge driven transport into cartilage and drug delivery for treating early stage post-traumatic osteoarthritis. Biomaterials.

[43] Hanne NJ, Easter ED, Cole JH (2019). Minimally invasive laser Doppler flowmetry is suitable for serial bone perfusion measurements in mice. Bone Reports.

[44] Ngo L, Knothe Tate ML (2020). New strategies for transport and drug delivery across length scales for osteoarthritis. ACS Biomat..

[45] Ngo, L. & Knothe Tate, M. L. (2023). Multi-modal sample preparation and imaging protocol for nano-to-mesoscopic mapping of cellular inhabitants in diverse tissue compartments, across organ systems. 30 May 2023, PROTOCOL (Version 1) available at Protocol Exchange 10.21203/rs.3.pex-2244/v1.

[46] Knothe Tate ML, Knothe UR (2000). An ex vivo model to study transport processes and fluid flow in loaded bone. J. Biomech..

[47] Knothe Tate ML, Steck R, Anderson EJ (2009). Bone as an inspiration for a novel class of mechanoactive materials. Biomaterials.

[48] Knothe Tate ML, Tami AEE, Netrebko P, Milz S, Docheva D (2012). Multiscale computational and experimental approaches to elucidate bone and ligament mechanobiology using the ulna-radius-interosseous membrane construct as a model system. Technol. Heal. Care.

[49] Wilson, D.L., Gargesha, M., Roy, D., Qutaish, M.Q., Krishnamurthi, G., Sullivant, K., Wuttisarnwattana, P., Lu, H., Moore, B., & Anderson, C. Cryo-imaging of 70+GB mice: Image processing/visualization challenges and biotechnology applications. in *2011 IEEE International Symposium on Biomedical Imaging: From Nano to Macro* 224–229 (IEEE, 2011). doi:10.1109/ISBI.2011.5872393.

